# Determining sample size in a personalized randomized controlled (PRACTical) trial

**DOI:** 10.1002/sim.10168

**Published:** 2024-07-09

**Authors:** Rebecca M. Turner, Kim May Lee, A. Sarah Walker, Sally Ellis, Michael Sharland, Julia A. Bielicki, Wolfgang Stöhr, Ian R. White

**Affiliations:** 1 MRC Clinical Trials Unit at University College London, London, UK; 2 Institute of Psychiatry, King’s College London, London, UK; 3 Global Antibiotic Research & Development Partnership (GARDP), Geneva, Switzerland; 4 Institute of Infection and Immunity, St. George’s University of London, London, UK; 5 University of Basel Children’s Hospital, Basel, Switzerland

**Keywords:** clinical trials, multiple treatments, personalized randomization, sample size, trial design

## Abstract

In clinical settings with no commonly accepted standard-of-care, multiple treatment regimens are potentially useful, but some treatments may not be appropriate for some patients. A personalized randomized controlled trial (PRACTical) design has been proposed for this setting. For a network of treatments, each patient is randomized only among treatments which are appropriate for them. The aim is to produce treatment rankings that can inform clinical decisions about treatment choices for individual patients. Here we propose methods for determining sample size in a PRACTical design, since standard power-based methods are not applicable. We derive a sample size by evaluating information gained from trials of varying sizes. For a binary outcome, we quantify how many adverse outcomes would be prevented by choosing the top-ranked treatment for each patient based on trial results rather than choosing a random treatment from the appropriate personalized randomization list. In simulations, we evaluate three performance measures: mean reduction in adverse outcomes using sample information, proportion of simulated patients for whom the top-ranked treatment performed as well or almost as well as the best appropriate treatment, and proportion of simulated trials in which the top-ranked treatment performed better than a randomly chosen treatment. We apply the methods to a trial evaluating eight different combination antibiotic regimens for neonatal sepsis (NeoSep1), in which a PRACTical design addresses varying patterns of antibiotic choice based on disease characteristics and resistance. Our proposed approach produces results that are more relevant to complex decision making by clinicians and policy makers.

## 1 Introduction

For some diseases, multiple treatment regimens are potentially useful for treatment, but particular regimens are contra-indicated for certain subgroups of patients and there is no standard of care regimen that would be appropriate for all, or even most, patients. Designing a randomized controlled trial to address the clinical questions of most relevance is challenging in these settings. Recruiting a sufficient number of patients to a conventional multi-arm randomized trial would be very difficult, because many available patients would be excluded for having contra-indications to one or more regimens, and any regimen designated “standard-of-care” by those designing the trial may not be considered standard or even acceptable by other investigators. When multiple regimens are under consideration and there is clinical uncertainty over which to use, a two-arm trial would not address the research questions of primary importance and may still have difficulties recruiting enough patients.

For example, there is substantial clinical uncertainty over which regimens to use for treating multi-drug-resistant infections or those with a considerable proportion of disease episodes expected to be caused by multidrug-resistant bacteria, resulting in an urgent need for randomized trials to provide comparisons of the treatment options available. In the area of carbapenem-resistant bacterial infections, treatment choices include high-dose carbapenems, older, potentially toxic drugs, newer drugs and combinations of drugs, and there is no consensus over which regimens should be used.^1–5^ Recruiting patients to a multi-arm trial comparing these regimens is problematic, because many patients with carbapenem-resistant infections have one or multiple contra-indications. Causes of contra-indications include the antimicrobial susceptibility of the infecting organism or the patient’s medical history/underlying disease, allergies or conditions such as impaired renal function. These issues make it difficult to find even two specific regimens to which large numbers of patients could be randomized, meaning that few randomized trials have been carried out to define optimal treatment.^6^

In a trial evaluating treatments for multi-drug-resistant infections, we want to compare multiple treatments to which different sets of patients can be randomized and obtain treatment rankings to inform clinical decisions. The data analysis required is analogous to a network meta-analysis,^7,8^ in which multiple treatments are compared in separate trials and the aim of the evidence synthesis is to rank treatments. We have previously proposed a new trial design for this setting, called a “Personalized Randomized Controlled Trial” (PRACTical) design.^6^ For a network of treatments to be compared, each patient recruited would be randomized only among regimens which are appropriate for that patient given their characteristics: we call this personalized randomization list their treatment “pattern.”

Standard methods for determining sample size in clinical trials are based on power to detect (superiority) or exclude (non-inferiority) a clinically relevant difference between two treatment arms under specific control of Type I and II error and are not applicable to a network of treatments, where the objective is ranking. In this paper we propose methods for determining sample size by examining how much information is provided by a PRACTical trial of a given sample size. As measures of trial information, we consider how much mortality would be prevented by choosing top-ranked treatments based on sample information and the probability that the top-ranked treatment is the best or nearly best treatment. The methods are applied to a motivating example of a PRACTical trial evaluating antibiotic regimens for neonatal sepsis. This trial incorporates the use of a Sequential Multiple Assignment Randomized Trial (SMART) design to allow randomization to second-line treatments where required (non-response to or failure of first-line antibiotics). We initially present methods for determining sample size for a PRACTical design requiring only one randomization, then present methods for determining sample size in a PRACTical SMART design, and finally apply both methods to our motivating example trial.

The layout of the paper is as follows. In section 2, we discuss our motivating example trial. Methods for determining sample size in a standard PRACTical design are described in section 3 and applied to the example trial in section 4. Methods for determining sample size in a PRACTical SMART design are proposed in section 5 and applied to the example in section 6. We conclude with a discussion in section 7.

## 2 Motivating Example: Neosep1

Our motivating example is a trial evaluating combination antibiotic regimens for neonatal sepsis. Neonatal sepsis causes a high burden of disease globally, particularly in low and middle income countries, with an estimated 1.3 (95% CI 0.8 to 2.3) million annual incident cases worldwide,^9^ resulting in 203 000 (95% CI 178 700 to 267 100) sepsis-attributable deaths.^10^ Increasing rates of resistance are reported globally and threaten to undermine the effectiveness of the current WHO recommended antibiotic regimens. The NeoSep1 trial was designed to evaluate novel combinations of older off-patent antibiotics for treatment of neonatal sepsis in comparison with WHO recommended and currently used regimens, and is sponsored and funded by the Global Antibiotic Research & Development partnership (GARDP).

A wide range of study designs were considered for the NeoSep1 trial, including conventional multi-arm designs and multi-arm multi-stage (MAMS) designs. However, these designs would be difficult to recruit to, since varying patterns of resistance and acceptability mean there would be no single standard of care arm to which multiple sites could randomize and many sites would struggle to randomize to all arms. A PRACTical design was chosen to allow multi-regimen comparisons to be made from sites randomizing across different sets of regimens, with infants randomized only to regimens considered clinically acceptable for that infant. The objective of the trial is to rank the novel and existing antibiotic regimens according to their clinical effectiveness, safety, cost and selection for resistance.

In neonatal sepsis, it is common for first-line treatment to fail and for infants to require second-line treatment. The design of NeoSep1 therefore also incorporates the use of SMART^11^ to allow randomization to second-line treatment where required; personalized randomization lists will be used for both randomizations.

At the first randomization, personalized randomization lists will be drawn from a list of 8 regimens (Table 1), while at the second randomization, personalized randomization lists will be determined by the infant’s first randomized regimen (Table 2). Under the current WHO guidelines, the recommended regimens are ampicillin+gentamicin (first-line treatment) and ceftriaxone/cefotaxime (second-line treatment). NeoSep1 will make comparisons between the WHO-recommended regimens, three other existing regimens (chosen from piperacillin/tazobactam, piperacillin/tazobactam+amikacin, ceftazidime, ceftazidime+amikacin and meropenem as part of site selection and set-up) and three novel regimens (fosfomycin+amikacin, fosfomycin+flomoxef and flomoxef+amikacin), when used as either first-line or second-line treatment for neonatal sepsis. The primary endpoint of the trial is 28-day mortality.

## 3 Methods For Determining Sample Size In A Practical Design

In a PRACTical design evaluating a network of treatments, each patient is randomized only between those treatments that are judged to be clinically acceptable for that patient. Suppose that a PRACTical trial comparing treatments *j* = *A, B, C*, … is planned. Suppose that patient 1 is eligible for randomisation to any of treatments A, B, D and H; these treatments then form patient 1’s personalized randomization list. If patient 2 is eligible for any of treatments C, E, F, G and J, these treatments form patient 2’s personalized randomization list. The overall trial could include patients with many different personalized randomization lists. In the analysis of a PRACTical design, we combine information about treatment effects across different types of patients using an approach similar to network meta-analysis, using indirect as well as direct evidence.^12^ More details about the method for analysis are given below. The primary aim of a PRACTical trial is to produce a set of treatment rankings that can guide choice of treatment for individual patients, with the overall aim of choosing one of the best treatments for each patient (or at least avoiding the worst treatments for each patient) rather than necessarily identifying the very best treatment.

In a PRACTical trial, standard power-based methods for determining sample size are not applicable, because the objective of the trial is to rank treatments rather than to test a null hypothesis. Instead, we choose an appropriate sample size by considering how much information would be gained by running trials of varying sizes. In a trial with a binary outcome, the value of trial information can be measured in terms of how many adverse outcomes (eg, deaths) could be prevented by choosing the top-ranked treatment for each patient based on the trial’s results rather than choosing a random treatment from the appropriate personalized randomization list before the trial.

We perform a simulation study based on the planned trial design and covering several plausible scenarios for expected frequencies of the primary outcome across treatments. In sample size calculations for a PRACTical design, the expected outcome frequencies across treatments play an equivalent role to that of the target treatment effect expected under the alternative hypothesis in a conventional sample size calculation for a superiority trial. For each scenario, we evaluate three different performance measures: mean reduction in adverse outcomes from using sample information, proportion of patients for whom the top-ranked treatment was within 2% or 1% (with respect to adverse outcome frequency) of the best treatment, and the proportion of patients for whom the top-ranked regimen was better than a randomly chosen treatment.

The general approach taken is as follows. Suppose that a PRACTical trial comparing treatments *j* = *A, B, C*, …, is planned. We use “pattern” to refer to the set of acceptable treatments for a patient and we refer to the set of patients with the same pattern as a “subgroup.” Let a pattern be *S_k_* ⊆ (*A, B, C*, …) for subgroup *k* = 1, …, *K*. In the sample size calculations, we need to make an assumption about which patterns *S_k_* will be present in the planned trial and about the likely frequencies *λ_k_* of each pattern *S_k_*. We will obtain clinical opinion about the expected probability of an adverse outcome under each of the treatments, denoted *P_jk_* for patients receiving treatment *j* in subgroup *k*, and use these to construct a set of plausible scenarios for the simulation study.

A range of possible sample sizes for the trial is determined. For each scenario, a large number of simulated trial data sets are generated for each sample size under consideration. The simulated trial data sets are analyzed using the planned method of analysis and the treatment rankings are determined.

### 3.1 Analysis of simulated data

As an appropriate approach for analyzing the data, we will use a network-based analysis that respects the PRAC-Tical randomization by controlling for pattern in the analysis model and produces a single treatment ranking for all patterns.^12^ Specifically, we will assume the following logistic model for analyzing binary outcomes, adjusting for pattern:

(1) $$logit\left(P_{jk}\right)=\alpha_{k}+\theta_{j}$$

where *α_k_* represents the log odds for the baseline risk of an adverse outcome for patients in subgroup *k* when treated with a pattern-specific reference treatment *j*′ (which depends on *k*, but we write *j*′ rather than *j*′(*k*) for simplicity), and *θ_j_* represents the log odds ratio for treatment *j* compared with treatment *j*′, with *θ_j_*′ = 0.

### 3.2 Performance measures

Following the analysis of each simulated trial data set, we calculate three performance measures that evaluate trial information by considering the estimated best treatments for each pattern. Let ${\hat{j}}_{k}=argmin_{j\in S_{k}}{\hat{P}}_{jk}$ represent the estimated best treatment in pattern *S_k_* based on analysis of the trial data, that is, the treatment that has the lowest estimated adverse outcome probability ${\hat{P}}_{jk}$.

- **Reduction in adverse outcomes**: We first quantify the reduction in an adverse outcome if treatment of a future population is informed by the results of the trial, in comparison to being treated using no information. We assume that treatment using no information is equivalent to making a random choice among each patient’s acceptable treatments, and assume also that the future distribution of subgroups will be the same as in the trial. We therefore define the reduction in adverse outcomes resulting from using the estimated best treatments by:
  $$R_{{\hat{j}}_{k}}=E\left[\underset{k}{\sum }{\hat{\lambda }}_{k}\left(mean_{j\in S_{k}}P_{jk}-P_{{\hat{j}}_{k}k}\right)\right]$$
  where ${\hat{\lambda }}_{k}$ is the observed prevalence of subgroup *S_k_* in the population, and the term in round brackets is the reduction in adverse outcomes from using the estimated best treatment in *S_k_* rather than the mean of true mortality rates across all eligible treatments within *S_k_*. To make interpretation easier, we will present the reduction in adverse outcomes as a percentage of the reduction which could be achieved if the true rankings of treatments were known:
  $$100\left\{R_{{\hat{j}}_{k}}/E\left[\underset{k}{\sum }{\hat{\lambda }}_{k}\left(mean_{j\in S_{k}}P_{jk}-\min_{j\in S_{k}}P_{jk}\right)\right]\right\}$$
  where $min_{j\in S_{k}}P_{jk}$ represents the probability of an adverse outcome under the truly best treatment in *S_k_*.
- **Near-best treatment probability**: Next, we examine the expected proportion of patients for whom the estimated best treatment is no more than *κ*% worse than the truly best treatment among their acceptable treatments. This is defined by:
  $$E\left[\underset{k}{\sum }{\hat{\lambda }}_{k}I\left(P_{{\hat{j}}_{k}k}\leq min_{j\in S_{k}}P_{jk}+\kappa \right)\right]$$
  where *I* denotes an indicator function.
- **Improvement over random choice**: We examine also the proportion of patients for whom the estimated best treatment is better than a treatment chosen randomly among their acceptable treatments:
  $$E\left[\underset{k}{\sum }{\hat{\lambda }}_{k}I\left(P_{{\hat{j}}_{k}k}\leq mean_{j\in S_{k}}P_{jk}\right)\right]$$

By averaging the above performance measures across simulated data sets, we can summarize the information provided by trials of a certain sample size. An appropriate sample size can then be chosen on the basis of its overall performance with respect to potential for preventing adverse outcomes.

## 4 Application To Neosep1 First-Line Treatments (Practical Design)

We will demonstrate how to determine sample size in a PRACTical design through applying our methods only to the first-line treatments in the NeoSep1 trial. At the first randomization, personalized randomization lists will be drawn from a list of 8 regimens, for example: ampicillin+gentamicin (A), ceftriaxone/cefotaxime (B), fosfomycin+amikacin (C), fosfomycin+flomoxef (D), flomoxef+amikacin (E), piperacillin/tazobactam (F), piperacillin/tazobactam+amikacin (G) and meropenem (H). Personalized randomization lists were expected to vary across geographical regions according to levels of antibiotic resistance and treatment costs. Investigators expected that three different personalized randomization lists or patterns would be used in the trial. Infants would be randomized between regimens A to E (Pattern 1) in countries which are predominantly still using WHO recommended regimens, between regimens C to H (Pattern 2) in countries where rates of resistance are moderate, or between regimens E, F and H (Pattern 3) in countries where rates of resistance are high (Table 1). We made two different assumptions about frequencies of patterns: equal pattern frequencies (equal split between Patterns 1-3) or unequal pattern frequencies (50% Pattern 1, 40% Pattern 2, 10% Pattern 3).

The primary endpoint of the NeoSep1 trial is 28-day mortality. Following treatment under the regimens available, based on data from the recently completed global neonatal sepsis cohort study NeoOBS^13^ it was considered plausible that 28-day mortality will vary from 10% to 20% across regimens. Fixed values for regimen effects were selected to achieve this variation (Table 1). This was our base case scenario. To examine the sensitivity of the performance measures to the assumptions made, we examined scenarios with larger treatment effects (log odds ratios increased by 25%) or smaller treatment effects (log odds ratios decreased by 25%) and we also used a reversed set of treatment effects as an extremely different scenario (Table 2). To explore the impact of changing the amount of within-pattern information available, we examined a scenario including an additional Pattern 4 in which any of the 8 regimens are acceptable, and a sparse scenario including fewer treatments per pattern (regimens A, B and E in Pattern 1, regimens C, D, E and G in Pattern 2, regimens E, F and H in Pattern 3). We generated 1000 simulated trial data sets for each scenario. The code is provided in supplementary material.

**Figure 1:**
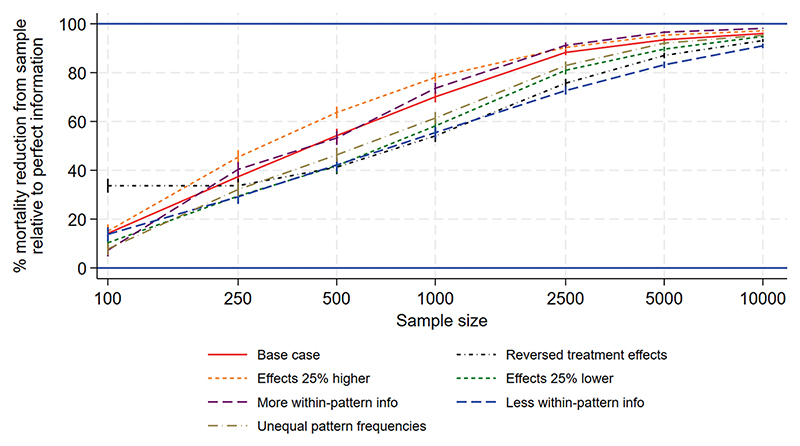
NeoSep1 first-line treatments: Mean reduction in % mortality from using the chosen treatment based on sample information rather than a randomly chosen treatment, as a percentage of reduction from perfect information.

**Figure 2:**
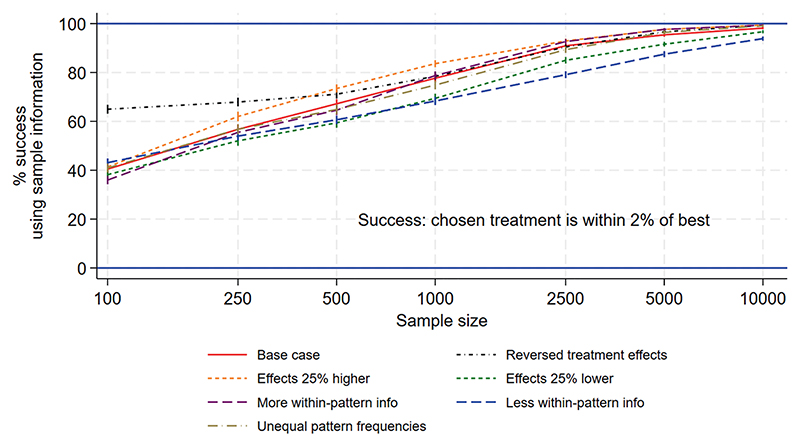
NeoSep1 first-line treatments: Frequency with which the chosen treatment based on sample information was within 2% (in mortality level) of the best treatment.

**Figure 3:**
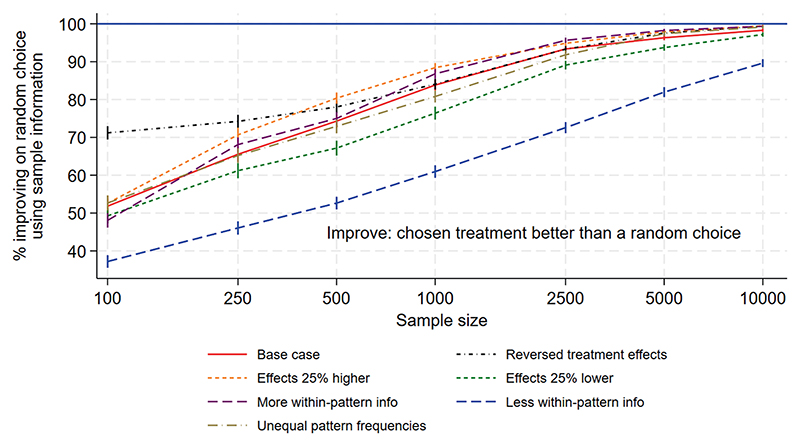
NeoSep1 first-line treatments: Frequency with which the chosen treatment based on sample information was better than a randomly chosen treatment.

Figures 1 to 3 show the performance measures for sample sizes between 100 and 10 000 under the base case scenario (patterns and regimen effects as shown in Table 1, equal pattern frequencies) and the other scenarios explored. Figure 1 examines the reduction in mortality that would be achieved if treatment of a future population is informed by the results of a trial rather than treated using no information. Figure 2 shows the frequency with which the mortality under the chosen treatment based on sample information would be within 2% of the mortality under the best treatment. Figure 3 shows the frequency with which the chosen treatment based on sample information performed better than a randomly chosen treatment. Using top-ranked treatments from a trial of size 100 would produce only 14% of the maximum possible mortality reduction based on perfect information under the base case scenario, while a trial of size 10 000 would achieve 96% of the maximum possible reduction (Figure 1). A trial including 100 patients would provide a 40% chance of the difference between mortality under the top-ranked and best treatments being less than 2%, while a trial of size 10 000 would provide a 98% chance (Figure 2). A trial of size 100 would provide a 52% chance that a top-ranked treatment is better than a randomly chosen treatment, while a trial of size 10 000 would provide a 98% chance.

Larger treatment effects are easier to detect and therefore all performance measures were higher than under the base case, for a given sample size. This means a smaller sample size would be required to achieve a chosen value of the performance measure, in comparison to the base case. Similarly, smaller treatment effects are more difficult to detect and all performance measures were lower than under the base case, meaning that higher sample sizes would be required. For the scenario including more within-pattern information, performance measures were similar to those under the base case. For the sparse scenario including fewer treatments per pattern, all performance measures were lower than under the base case and the chance of a top-ranked treatment being better than a random choice was substantially lower (Figure 3). The performance measures were very different for the scenario with reversed treatment effects in comparison with the base case: the curves were flatter and increased more slowly with sample size. Assuming unequal rather than equal pattern frequencies made little difference to most performance measures, although the mortality reduction that would be achieved was slightly lower than under the base case (Figure 1).

## 5 Methods For Determining Sample Size In A Practical Smart Design

We now extend the methods presented in section 3 to incorporate personalized randomization to second-line treatment in a PRACTical SMART design. As for a PRACTical design without SMART randomization, we choose an appropriate sample size by considering how much information would be gained by running trials of varying sizes, using the same performance measures as previously. Since we want to evaluate how decisions made for future patients would be affected by all information obtained from the trial, we consider the information gained from ranking strategies comprising first-line and second-line treatment (eg, cefotaxime followed by meropenem) rather than the information gained from ranking first-line and second-line treatments separately.

The general approach taken is as follows. Suppose that a PRACTical SMART trial is planned, where patients will be randomized between some of treatments *j* = *A*_1_, *B*_1_, *C*_1_, … as first-line treatment. Let *q_jk_* be the probability that patients treated with treatment *j* in subgroup *k* need second-line treatment, where *q_jk_* could be assumed equal or varying across treatments and subgroups. Where second-line treatment is necessary, we assume that patients are then randomised between some of treatments *l* = *A*_2_, *B*_2_, *C*_2_, … as second-line treatment.

As previously we use pattern to refer to the set of acceptable treatments for a patient and we refer to the set of patients with the same pattern as a subgroup. We denote the first-line treatment patterns as *S*_1*k*_ ⊆ {*A*_1_, *B*_1_, *C*_1_, …} for subgroups *k* = 1, …, *K*. When second-line treatment is necessary, we denote second-line treatment patterns as *S*_2*m*_ ⊆ {*A*_2_, *B*_2_, *C*_2_, …} for subgroups *m* = 1, …, *M*; *S*_2*m*_ will depend on the first-line treatment received, for example because the treatment already given will be excluded from *S*_2*m*_. In the sample size calculations we need to make an assumption about which first-line and second-line treatment patterns will be present in the planned trial and about the likely frequencies *λ*_1*k*_, *λ*_2*m*_ of patterns *S*_1*k*_, *S*_2*m*_ respectively. We obtain clinical opinion about the expected probability of an adverse outcome under each of the treatments. We denote the expected probabilities as *P_jklm_* for patients receiving first-line treatment *j* in subgroup *k* followed by second-line treatment *l* in subgroup *m*. These are used to construct a set of plausible scenarios for the simulation study.

As described in section 3, we then determine a range of possible sample sizes for the trial and generate a large number of simulated trial data sets for each sample size under consideration, for each scenario. The simulated trial data sets are analyzed using the planned method of analysis and the treatment rankings are determined.

### 5.1 Analysis of simulated data

We extend model (1) to allow for PRACTical randomization to second-line treatments by controlling for second-line as well as first-line treatment patterns in the analysis model. Specifically, we assume the following logistic model for analyzing binary outcomes:

(2 $$logit\left(P_{jklm}\right)=\alpha_{km}+\theta_{j}+\psi_{l}$$

where *α_km_* represents the log odds for the baseline risk of an adverse outcome for a patient in subgroups *k, m* when treated with reference treatments *j*′, *l*′, *θ_j_* represents the log odds ratio for treatment *j* compared with treatment *j*′, with *θ_j_*′ = 0, and *ψ_l_* represents the log odds ratio for treatment *l* compared with treatment *l*′, with *ψ_l_*′ = 0.

**Figure 4:**
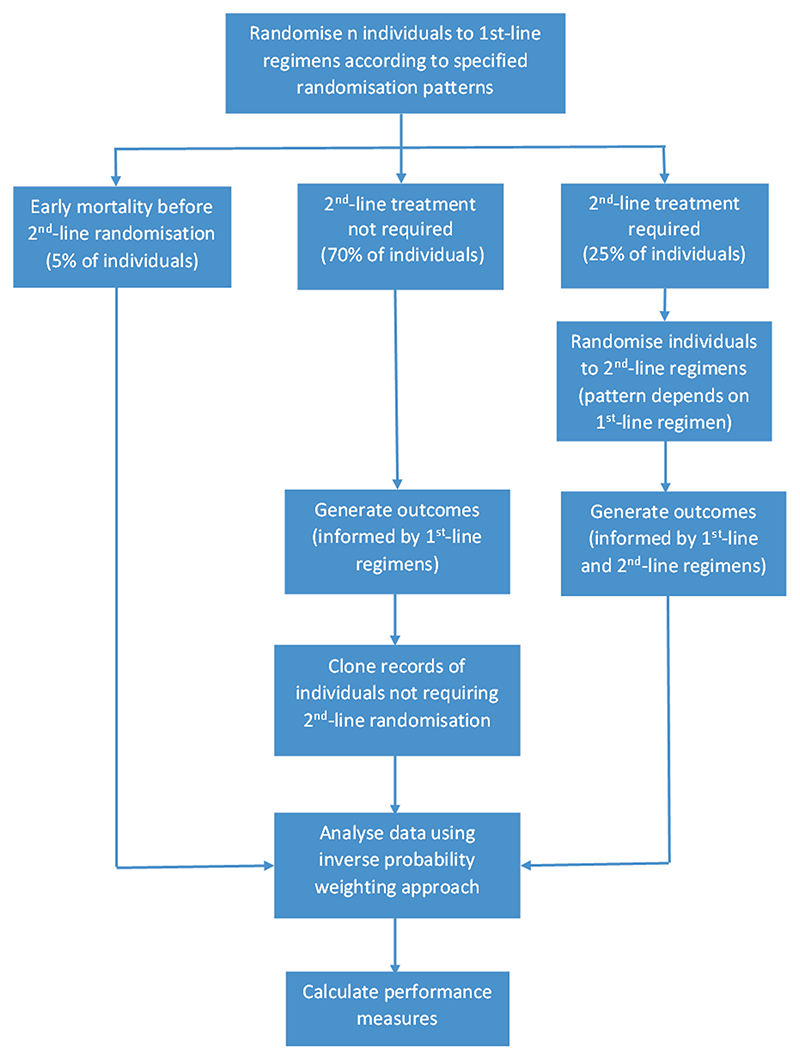
Illustration of process for simulating and analyzing data under a PRACTical SMART design.

In our analysis, we need to consider how to handle simulated individuals who did not require a second randomization. Our interest is in marginal treatment effects, averaged over those who did or did not require second-line treatment, rather than in treatment effects that are conditional on requiring second-line treatment. For this reason, we use an inverse probability weighting approach to allow us to estimate marginal treatment effects across the entire population. This involves assuming in the simulations that all individuals have a hypothetical second randomization. For simulated individuals who did not require a second randomization (including those who experienced early mortality), we created cloned records to represent all the regimens they could have received if randomized a second time, and then generated potential outcomes they could have experienced. An inverse probability weighting approach has been taken in analysis, where each cloned record was allocated a weight equal to 1/(number of treatments in second-line treatment pattern). This choice of weights ensures that each unswitched individual has the same weight in the analysis (summed over their cloned records) as each switched individual. This approach enabled the performance measures described below to be calculated for all simulated individuals. The process of simulating and analyzing data under a PRACTical SMART design is illustrated in Figure 4.

### 5.2 Performance measures

In order to evaluate how decisions made for future patients would be informed by all information obtained from the trial, we define performance with respect to chosen first-line/second-line treatment strategies (ie, combinations of first-line and second-line treatments) rather than considering first-line and second-line treatments separately. Let $\left({\hat{j}}_{k},{\hat{j}}_{m}\right)=argmin_{j\in S_{1\mathrm{k,}}}l\in S_{2m}{\hat{P}}_{jklm}$ represent the estimated best first-line/second-line treatment strategy for a patient with first-line treatment pattern *k* and second-line treatment pattern *m*.

- **Reduction in adverse outcomes**: We first quantify the reduction in an adverse outcome if treatment of a future population is informed by the results of the trial, in comparison to being treated using no information. We assume that treatment using no information is equivalent to making a random choice among each patient’s acceptable treatment strategies, and assume also that the future distribution of subgroups will be the same as in the trial. We therefore define the reduction in adverse outcomes resulting from using the estimated best treatments by:
  $$R_{{\hat{j}}_{k}{\hat{j}}_{m}}=E\left[\underset{k,m}{\sum }{\hat{\lambda }}_{km}\left(mean_{j\in S_{1k},l\in S_{2m}}P_{jklm}-P_{{\hat{j}}_{k}k{\hat{j}}_{m}m}\right)\right]$$
  where ${\hat{\lambda }}_{km}$ is the observed prevalence of the intersection of subgroups *S*_1*k*_ and *S*_2*m*_ in the population, and the term in round brackets is the reduction in adverse outcomes from using the estimated best treatment strategy rather than the mean of true mortality rates across all eligible treatment strategies. To make interpretation easier, we will present the reduction in adverse outcomes as a percentage of the reduction could be achieved if the true rankings of treatments were known:
  $$100\left\{R_{{\hat{j}}_{k}{\hat{j}}_{m}}/E\left[\underset{k,m}{\sum }{\hat{\lambda }}_{km}\left(mean_{j\in S_{1\mathrm{k,}}l\in S_{2m}}P_{jklm}-min_{j\in S_{1\mathrm{k,}}l\in S_{2m}}P_{jklm}\right)\right]\right\}$$
  where $min_{j\in S_{k},l\in S_{m}}P_{jklm}$ represents the probability of an adverse outcome under the truly best treatment strategy for patients in subgroups *S*_1*k*_ and *S*_2*m*_.
- **Near-best treatment probability**: Next we examine the expected proportion of patients for whom the estimated best treatment strategy is no more than *κ*% worse than the truly best treatment strategy among their acceptable treatment strategies. This is defined by:
  $$E\left[\underset{k,m}{\sum }{\hat{\lambda }}_{km}I\left(P_{{\hat{j}}_{k}\hat{k}{\hat{j}}_{m}m}\leq min_{j\in S_{1k},l\in S_{2m}}P_{jklm}+\kappa \right)\right]$$
  where *I* denotes an indicator function.
- **Improvement over random choice**: We examine also the proportion of patients for whom the estimated best treatment strategy is better than a treatment strategy chosen randomly among their acceptable treatment strategies:
  $$E\left[\underset{k,m}{\sum }{\hat{\lambda }}_{km}I\left(P_{{\hat{j}}_{k}\hat{k}{\hat{j}}_{m}m}\leq mean_{j\in S_{1\mathrm{k,}}l\in S_{2m}}P_{jklm}\right)\right]$$

By averaging the above performance measures across simulated data sets, we can summarize the information provided by trials of a certain sample size. An appropriate sample size can then be chosen on the basis of its overall performance with respect to potential for preventing adverse outcomes.

## 6 Application To Neosep1 (Practical Smart Design)

To carry out sample size calculations for the NeoSep1 trial, we needed to make assumptions about expected frequencies of randomization patterns, treatment effects and the percentage of infants switching to second-line treatment. As in section 4, we assumed that for first-line treatment infants would be randomized between regimens A to E (Pattern 1) in countries where WHO-recommended regimens are still commonly used, between regimens C to H (Pattern 2) in countries with moderate rates of resistance, or between regimens E, F and H (Pattern 3) in countries with high rates of resistance. We made two different assumptions about frequencies of patterns: equal pattern frequencies (equal split between Patterns 1-3) or unequal pattern frequencies (50% Pattern 1, 40% Pattern 2, 10% Pattern 3). At the second randomization, personalized randomization lists are determined by the patient’s first randomized regimen (Table 3), with an additional restriction that regimens C, D and G (containing amikacin) are unsuitable for infants randomized under Pattern 3 initially (who are living in countries with high rates of resistance). When analyzing the simulated data, this means that *α_km_* in (2) becomes *α_k_*.

The primary endpoint of the NeoSep1 trial is 28-day mortality. Following treatment under the first-line/second-line treatment strategies available, 28-day mortality is expected to vary from 10% to 20% across strategies (Table 3). In the sample size calculations, fixed values for first-line and second-line regimen effects have been selected to achieve this variation: a set of regimen effects (Table 4) was chosen through experimentation to provide a set of expected mortality values varying from 10% to 20% (Table 3). We needed to make allowance for expected levels of early mortality before second randomization and have assumed this to be 5%, with no variation across regimens. In the absence of early mortality, the percentage of infants switching to a randomized second-line treatment was expected to be between 25% and 50% across all treatments and patterns in the NeoSep1 trial. For the purpose of exploring the performance measures under a wider range of switching scenarios in this paper, we have additionally looked at scenarios assuming 10% or 75% switching, that is, we assumed *q_jk_* to be 0.1, 0.25, 0.5 or 0.75 for all *j, k*. In total, we examined five data generating scenarios: a base case with patterns and regimen effects as shown in Table 3, equal pattern frequencies and 25% switching to second-line treatment; a scenario with unequal pattern frequencies; and scenarios with 10%, 50% or 75% switching to second-line treatment. Initially, we also looked at a scenario with an extremely different set of treatment effects (reversed from the initial values), but these results are not presented because the scenario is unrealistic for this trial.

**Figure 5:**
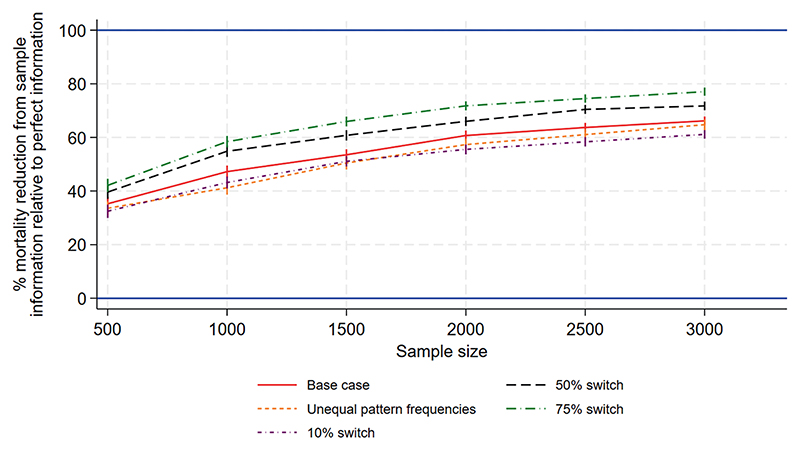
NeoSep1 SMART design: Mean reduction in % mortality from using the chosen treatment strategy based on sample information rather than a randomly chosen strategy, as a percentage of reduction from perfect information.

**Figure 6:**
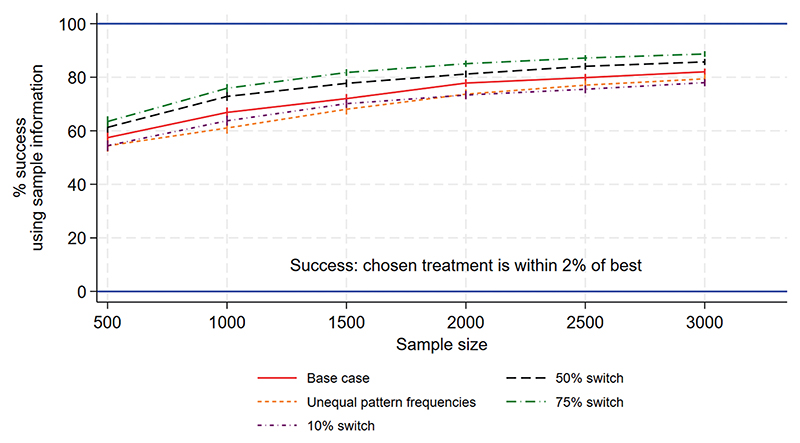
NeoSep1 SMART design: Frequency with which the chosen treatment strategy based on sample information was within 2% (in mortality level) of the best treatment strategy.

**Figure 7:**
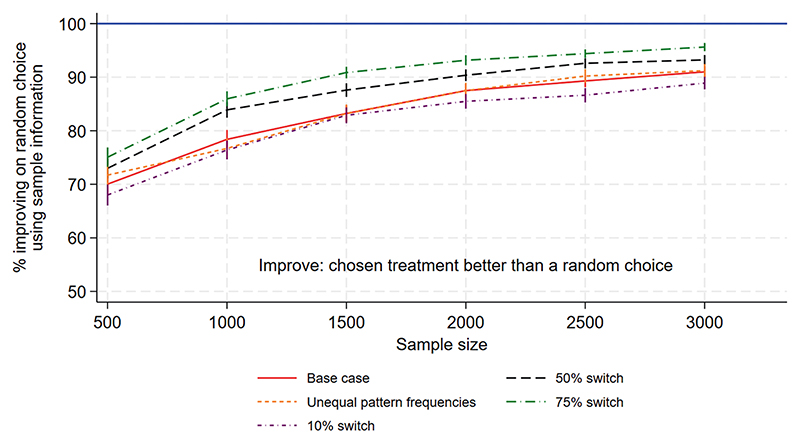
NeoSep1 SMART design: Frequency with which the chosen treatment strategy based on sample information was better than a randomly chosen strategy.

Figures 5 to 7 show the performance measures for sample sizes between 500 and 3000, which were considered to be realistic sample sizes for the NeoSep1 trial, under the five data generating scenarios. We first examine the reduction in mortality that would be achieved if treatment of a future population is informed by the results of a trial rather than treated using no information. Using top-ranked treatment strategies from a trial of size 500 would produce 34%−40% of the maximum possible mortality reduction based on perfect information (under the expected switching proportions of 25%−50%), while a trial of size 3000 would achieve at least 65% of the maximum possible reduction (Figure 5). A trial including 500 patients would provide 54%−61% chance of the difference between mortality under the top-ranked and best treatment strategies being less than 2% (Figure 6). On the other hand, a trial including 3000 patients would provide 79%−86% chance of the difference being less than 2%. A trial of size 500 provides a 70%−73% chance that a top-ranked treatment strategy is better than a randomly chosen strategy, while a trial of size 3000 provides a >90% chance (Figure 7).

Based on the results obtained for these performance measures, the NeoSep1 investigators decided to include 3000 infants in their trial. The simulations carried out relied heavily on assumptions about frequencies of randomization patterns, proportions of infants switching to second-line treatment and mortality values expected under each treatment strategy. To address the problem that the assumptions made might not be correct, the trial investigators plan to check the sample size after 50% follow-up has been achieved, using accrued evidence on the design parameters, to explore whether any modifications to sample size are required. Checking the variation in accrued endpoints across regimens will involve looking at unblinded data. Because the focus of a PRACTical design is on ranking treatments rather than hypothesis testing, we do not control type I error as in a conventional trial design, so there is no need to adjust the final analysis to make allowance for looking at interim data.

We explored a wider range of scenarios for numbers of patients switching to second-line treatment than those expected in the NeoSEP1 trial (Figures 5–7). We found that the amount of information provided by the trial increases if more patients switch to second-line treatment, meaning that if higher proportions are expected to switch, the trial would need to recruit a smaller sample size to achieve the same performance measures. However, the differences in performance measures are relatively small; for example, a trial including 1000 patients would provide a 76% chance of improving over a randomly chosen treatment strategy if 10% of patients switch to second-line treatments, or a 78%, 84% or 86% chance respectively if 25%, 50% or 75% of patients switch.

## 7 Discussion

When designing a trial evaluating a network of treatments, sample size cannot be determined using standard power-based methods, because the aim is to rank treatments rather than test a null hypothesis. We have proposed evaluating how much information would be provided by trials of differing sizes by considering how well the top-ranked treatment for each patient would perform in comparison to their optimal treatment or a randomly chosen treatment. The resulting plots are straightforward to interpret and facilitate discussion of sample size with clinical investigators. Arbitrary but commonly used thresholds of power (80%−90%) can be used to identify sample sizes which provide similar proportionate increases in information gained compared with the current status quo of essentially random choice from a personalized randomization list for each patient. We have demonstrated application of this approach to the NeoSep1 trial, initially for a design including the first-line treatments only, and then for the full design including both first-line and second-line treatments with SMART randomization.

The performance measures proposed in this paper are appropriate for designing trials with a binary outcome, but could be adapted for other outcome types. For a continuous outcome, performance measures could be based on mean differences or standardized mean differences between treatments; for example, we could examine the mean reduction in blood pressure resulting from using top-ranked treatments rather than randomly chosen treatments. For a time-to-event outcome, investigators would need to consider which metric would be most appropriate for summarizing the information from their trial. For example, we could examine the survival difference at 2 years resulting from using top-ranked treatments rather than randomly chosen treatments, or we could instead examine the difference in restricted mean survival time, which is a measure of average survival up to a specified time point.^14,15^

The methods could easily be extended to include planned adjustment in the trial analysis for baseline covariates, by adding these into the model used to analyze the simulated trial data. Sample size calculations should make allowance for anticipated levels of missing data; within our approach, this could either be done by modelling missingness when simulating trial data or in a standard way by inflating the chosen sample size by an appropriate amount. When specifying performance measures, we assumed that having no trial information is equivalent to making a random choice between acceptable treatments. Other choices may be more appropriate in particular settings and performance measures could be modified to accommodate these; for example, having no trial information might cause clinicians to choose the cheapest acceptable treatment.

Our proposed approach to determining sample size is informed by simulations, so this approach is much more time-consuming than a conventional power-based calculation. As in a standard sample size calculation, an assumption about expected treatment effects is required, but for a PRACTical design we also need to make assumptions about expected randomization patterns and pattern frequencies. We suggest performing sensitivity analyses to check the robustness of the performance measures to the assumptions made, as demonstrated in this paper. In our simulation study, we found that configuration and magnitude of treatment effects was very influential, as was the number of treatments per pattern, while a change in pattern frequencies had less impact on the conclusions. When designing a PRACTical trial, it is therefore important to have some advance knowledge of the likely treatment effects and also the expected patterns of randomization between treatments. In the NeoSep1 trial, the assumed treatment effects were based on data from a previous observational study and expected randomization patterns were informed by knowledge of geographical resistance to certain antibiotic regimens. The NeoSep1 trial investigators plan to re-evaluate the planned sample size after 50% follow-up has been achieved, using updated estimates of treatment effects, randomization patterns and the proportion of infants requiring second-line treatment. In a PRACTical design, the focus is on ranking available treatments rather than testing for statistically significant treatment effects, so the potential impact of sample size re-estimation on type I error rates is not of concern here.

The additional assumptions and simulation-based methods required make determining sample size more challenging for a PRACTical design than for a conventional trial design. A PRACTical design should therefore be chosen only when necessary, that is in settings where multiple regimens are potentially useful but contra-indications for some subgroups mean that no there is no single standard of care regimen appropriate for all or most patients. In such settings, using a PRACTical design is likely to be the only way of running a randomized trial to which a sufficient number of patients can be recruited. In settings where multiple regimens are potentially useful but there is a standard of care regimen that would be appropriate for most patients, a multi-arm or multi-arm multi-stage design would be suitable and would provide efficiency gains over running a series of pairwise trials.^16^

Our approach to choosing sample size is related to Expected Value of Sample Information (EVSI) approaches for sample size determination,^17^ but we have considered the non-monetary rather than monetary benefits of running a clinical trial. An EVSI approach involves constructing a health economic model for the target population, that incorporates treatment costs and the societal costs incurred for patients experiencing different outcomes, to estimate the economic value of collecting data from a planned trial. In our approach, we calculated the expected value of sample information with respect to reduction of adverse outcomes. The Expected Value of Perfect Information (EVPI) is useful in EVSI calculations as an upper bound for the value of running a clinical trial^17^; we used this concept when presenting the reduction in adverse outcomes based on trial information as a percentage of the reduction achievable from perfect information.

For a PRACTical trial design including a network of treatments, we have proposed taking a value of information approach to determining sample size. We recommend examining several performance measures to evaluate the benefits of running trials of varying sizes, with respect to preventing adverse outcomes. The resulting plots are straightforward to interpret and should facilitate decision making for investigators designing trials in settings where multiple treatments are potentially useful, but some treatments are unacceptable for certain subgroups of patients.

## Supplementary Material

Code for running simulations

Supplementary figures

## Figures and Tables

**Table 1:** Randomization patterns under PRACTical design for NeoSep1 and assumed values for true mortality under each regimen (first-line treatments only).

Regimen	Pattern 1	Pattern 2	Pattern 3	OR for mortality (regimen vs Amp/Pen+Gent)	Assumed true mortality under regimen
Amp/Pen+Gent	Yes	No	No	1	20.0%
Cefotaxime	Yes	No	No	0.99	19.8%
Fos+Amik	Yes	Yes	No	0.84	17.4%
Flom+Amik	Yes	Yes	No	0.84	17.3%
Fos+Flom	Yes	Yes	Yes	0.81	16.9%
Pip-Taz	No	Yes	Yes	0.76	15.9%
Pip-Taz+Amik	No	Yes	No	0.70	15.0%
Meropenem	No	Yes	Yes	0.45	10.1%

**Table 2:** Assumed values for true mortality under each regimen (first-line treatments only) under modified scenarios used in sensitivity analyses.

Regimen	Larger treatment effects	Smaller treatment effects	Reversed treatment effects
Amp/Pen+Gent	20.0%	20.0%	10.1%
Cefotaxime	19.8%	19.9%	15.0%
Fos+Amik	16.8%	18.0%	15.9%
Flom+Amik	16.6%	17.9%	16.9%
Fos+Flom	16.1%	17.6%	17.3%
Pip-Taz	15.0%	16.9%	17.4%
Pip-Taz+Amik	13.9%	16.1%	19.8%
Meropenem	8.4%	12.1%	20.0%

**Table 3:** Randomization patterns at second randomization under PRACTical SMART design for NeoSep1 and assumed values for true mortality under each first-line/second-line treatment strategy.

	Regimens available at second randomization							
Regimen chosen at first randomization	Cef	Fos+ Amik	Flom+ Amik	Fos+ Flom	Pip-Taz	Pip-Taz+Amik	Mero	
Amp/Pen+Gent	Yes (19.7%)	Yes (17.7%)	Yes (17.3%)	Yes (17.1%)	Yes (16.9%)	Yes (15.0%)	No	
Cefotaxime	No	Yes (17.6%)	Yes (17.1%)	Yes (17.0%)	Yes (16.7%)	Yes (14.9%)	Yes (13.2%)	
Fos+Amik	No	No	Yes (15.0%)	No	Yes (14.6%)	Yes (12.9%)	Yes (11.4%)	
Flom+Amik	No	Yes (15.2%)	No	No	Yes (14.5%)	Yes (12.8%)	Yes (11.3%)	
Fos+Flom	No	No	No	No	Yes (14.1%)	Yes (12.5%)	Yes (11.0%)	
Pip-Taz	No	Yes (14.0%)	Yes (13.6%)	Yes (13.5%)	No	No	Yes (10.4%)	
Pip-Taz+Amik	No	Yes (13.2%)	Yes (12.8%)	Yes (12.7%)	No	No	Yes (9.7%)	
Meropenem^a^	No	No	No	No	No	No	No	

*Note*: The mortality values *P_jklm_* arise from an additive model (2) for first-line/second-line regimen effects on a logistic scale: *logit* (*P_jklm_*) = *α_km_* + *θ_j_* + *ψ_l_*, where *α_km_* = *logit*(0.20) represents the log odds for mortality under Amp/Pen+Gent; *θ_j_* and *ψ_l_* represent the first-line and second-line regimen effects (on log odds ratio scale) vs Amp/Pen+Gent (Table 4); *ψ_l_*=0 for individuals with no second randomisation.

a No second randomization. Assumed value for true mortality is 10.1% (see Table 1).

**Table 4:** Assumed values for regimen effects under PRACTical SMART design for NeoSEP1.

Regimen	OR for mortality as first-line treatment	OR for mortality as second-line treatment
Amp/Pen+Gent	1	1
Cefotaxime	0.99	0.98
Fos+Amik	0.84	0.86
Flom+Amik	0.84	0.84
Fos+Flom	0.81	0.83
Pip-Taz	0.76	0.81
Pip-Taz+Amik	0.70	0.70
Meropenem	0.45	0.61

## Data Availability

Code used for creating the simulated data sets is available in the supplementary material.
