# Supplementary material for: Determining sample size in a personalized randomized controlled (PRACTical) trial
Source: Stat Med. Author manuscript; Available in PMC 2024 Oct 1. (PMC7616655; doi:10.1002/sim.10168)
Supplement: Code for running simulations [file EMS198932-supplement-Code_for_running_simulations.pdf]

Code for running simulations

Stata do-file for simulating and analysing data for a PRACTical design

```
local name prt_ss
local patterns patternsfirstline_1
local nsims 100
local nobslist 100 250 500 1000 2500 5000 10000
local psi psi1
local margin 0.02
local run 1
set seed 578156
set trace off

if `run' {

cap postclose simstemp
postfile simstemp nobs i str8 method EY success improve using `name'_postfile,
replace

foreach nobs of local nobslist {
   di as input _new(2) "nobs=`nobs'"

   forvalues i=1/`nsims' {
      qui {

         * count treatments
         use `patterns', clear
         unab vars : canhave*
         local ntrts : word count `vars'

         * generate data structure and randomisation
         practical_gen, canhave(canhave) prob(prob) nobs(`nobs')
         sort randtrt

         * generate outcomes
         rename randtrt randtrt1
         merge m:1 randtrt1 using psi1
         gen py = invlogit(alpha + `psi')
         gen y = runiform() < py

         * analysis of data
         cap logit y i.randtrt1 i.pattern, iter(100)
         if _rc cap logit y ibn.randtrt1 i.pattern, nocons iter(100)
         if _rc {
            di as error "Non convergence at iteration `i'"
            continue
         }

         * make decisions
         gen EYsample = .
         gen bestpred = .
         gen trts=0
         gen EYperfect = .
         gen EYno=0
         gen trueprob = .

         * matrix of true probabilities
         matrix Q=.
         forvalues j=1/`ntrts' {
            summ py if randtrt1==`j'
            scalar trueprob=r(mean)
            matrix Q=Q\trueprob
         }
         matrix P=Q[2..`ntrts'+1,1]
```

```
        forvalues trt=1/`ntrts' {

           qui replace randtrt1 = `trt'
           qui predict pred, rules // handles perfect prediction
           qui replace trueprob = P[`trt',1]

           * using no information: random choice (sum true event probabilities for
available treatments in order to find average)
           qui replace trts = trts+1 if canhave`trt'
           qui replace EYno = EYno+trueprob if canhave`trt'

           * using perfect information
           qui replace EYperfect = min(EYperfect, trueprob) if canhave`trt'

           * using sample information
           qui replace EYsample = trueprob if pred<bestpred & canhave`trt'
           qui replace bestpred = pred if pred<bestpred & canhave`trt'

           drop pred
        }
        qui replace EYno=EYno/trts
        foreach method in no sample perfect {
           summ EY`method', meanonly
           local EYmean = r(mean)
           gen success`method' = EY`method' < EYperfect + `margin' + 0.0005
           summ success`method', meanonly
           local successprob = r(mean)
           gen improve`method' = EY`method' < EYno
           summ improve`method', meanonly
           local improveprob = r(mean)
           post simstemp (`nobs') (`i') ("`method'") (100*`EYmean')
(100*`successprob') (100*`improveprob')
        }
     }
   }

}
postclose simstemp
}

use `name'_postfile, clear
reshape wide EY success improve, i(i nobs) j(method) string
foreach method in perfect sample {
   gen gain`method' = EYno - EY`method'
}

* generate plots of results
local nobsmin .
local nobsmax .
foreach nobs of local nobslist {
   local nobsmin= min(`nobsmin',`nobs')
   local nobsmax= max(`nobsmax',`nobs')
}
local nobsmax=1.2*`nobsmax'

table nobs, statistic(mean gainperfect gainsample successsample improvesample)

local percentmargin=`margin'*100
   summ gainperfect, meanonly
   local gainperfect = r(mean)
   gen gainpercent = (gainsample/`gainperfect')*100
   meangr8 gainpercent nobs, name(g,replace) saving(g,replace) xlabel(`nobslist')
xscale(log) xscale(range(`nobsmin', `nobsmax')) ytitle("% mortality reduction from
sample information" "relative to perfect information") xtitle(Sample size)
color(red) yli(0 100, lcol(blue) lpattern(solid)) lwidth(*2) yscale(range(0 100))
ylabel(#6)

   meangr8 successsample nobs, name(s1,replace) saving(s1,replace)
xlabel(`nobslist') xscale(log) xscale(range(`nobsmin', `nobsmax')) ytitle("%
```

```
success" "using sample information") xtitle(Sample size) color(red) yli(0 100,
lcol(blue) lpattern(solid)) lwidth(*2) yscale(range(0 100)) note("Success: chosen
treatment is within `percentmargin'% of best.")

   meangr8 improvesample nobs, name(i,replace) saving(i,replace) xlabel(`nobslist')
xscale(log) xscale(range(`nobsmin', `nobsmax')) ytitle("% improving on random
choice" "using sample information") xtitle(Sample size) color(red) yli(50 100,
lcol(blue) lpattern(solid)) lwidth(*2) yscale(range(50 100)) note("Improve: chosen
treatment better than a random choice.")



save sims.dta, replace
```

Stata do-file for simulating and analysing data for a PRACTical SMART design

```
local name prtsmart_ss
local patterns1 patternsfirstline_1
local patterns2 patternssecondline
local nsims 100
local nobslist 500 1000 1500 2000 2500 3000
local pswitchlist 0.25 0.50
local psi1 psi1
local psi2 psi2
local meronum 8
local margin 0.02 // amount by which treatment may be inferior to best
local run 1
set seed 578156
set trace off

if `run' {

cap postclose simstemp
postfile simstemp str10 patterns1 pswitch nobs i str8 method EY success improve
using `name'_postfile, replace

foreach pswitch of local pswitchlist {
foreach nobs of local nobslist {
   di as input _new(2) "pswitch=`pswitch', nobs=`nobs'

   forvalues i=1/`nsims' {
      qui {

         * count treatments
         use `patterns1', clear
         unab vars : canhave*
         local ntrts : word count `vars'

         * generate data structure and first randomisation
         practical_gen, canhave(canhave) prob(prob) nobs(`nobs')
         rename canhave* firstcanhave*
         sort randtrt

         * early mortality (before second randomisation)
         gen z = runiform() < 0.05

         * second-line treatment required for proportion (=pswitch) of infants
         gen secondreq = runiform() < `pswitch' if z==0 & randtrt!=`meronum'
         replace secondreq = 0 if z==1 | randtrt==`meronum'

         * second randomisation
         merge m:1 randtrt using `patterns2'
         rename randtrt randtrt1
         * in NeoSep1, no AMIK treatments for those in pattern 3 for first
randomisation
         foreach j of numlist 3 4 7 {
            qui recode canhave`j' 1=0 if pattern==3
         }
```

```
        local ntrt 0
        local trtlist ""
        foreach var of varlist canhave* {
          local ++ntrt
          local trt = substr("`var'",length("canhave")+1,.)
          local trtlist `trtlist' `trt'
        }

        egen ncanhave=rsum(canhave*)
        gen randtrt2=.
        gen rand = runiform()
        foreach trt of local trtlist {
          replace randtrt2 = `trt' if randtrt2==. & canhave`trt' & rand<1/ncanhave
& secondreq==1
          replace rand=rand-1/ncanhave if canhave`trt'
        }
        drop rand

        * generate outcomes
        drop _merge
        merge m:1 randtrt1 using `psi1'
        drop _merge
        merge m:1 randtrt2 using `psi2'
        gen py = invlogit(alpha + psi1 + psi2) if z!=1
        replace py = invlogit(alpha + psi1) if psi2==.&z!=1
        gen y = runiform() < py | z==1

        * clone records where second randomisation not required
        sort randtrt1 randtrt2
        gen id=_n
        expand `ntrts'-1 if secondreq==0 & randtrt1!=`meronum'
        sort id
        by id: egen rank=rank(_n) if secondreq==0 & randtrt1!=`meronum'
        replace randtrt2=rank+1 if randtrt2==.&randtrt1!=`meronum'
        forvalues j=2/`ntrts' {
          drop if canhave`j'==0&randtrt2==`j'
        }
        by id: egen size=count(id)
        gen p=1/size
        rename canhave* secondcanhave*

        * analysis of data
        * if randtrt1 is meropenem, set randtrt2 to meronum+1 to represent no
second-line randomisation
        replace randtrt2=`meronum'+1 if randtrt1==`meronum'
        cap logit y i.randtrt1 i.randtrt2 i.pattern [pweight=1/p], vce(cluster id)
iter(100)
        if _rc cap logit y ibn.randtrt1 i.randtrt2 i.pattern [pweight=1/p], nocons
vce(cluster id) iter(100)
        if _rc {
          di as error "Non convergence at iteration `i'"
          continue
        }

        * make decisions
        gen EYsample = .
        gen bestpred = .
        gen trts=0
        gen EYperfect = .
        gen EYno=0
        gen trueprob = .
        gen worstprob = .

        * matrix of treatment strategies
        egen trtcomb=group(randtrt1 randtrt2)
        summ trtcomb
        local ntrtcombs=r(max)
        matrix S1=.
```

```
        matrix S2=.
        forvalues j=1/`ntrtcombs' {
          summ randtrt1 if trtcomb==`j'
          scalar treat1=r(mean)
          matrix S1=S1\treat1
          summ randtrt2 if trtcomb==`j'
          scalar treat2=r(mean)
          matrix S2=S2\treat2
          }
        matrix subS1=S1[2...,1]
        matrix subS2=S2[2...,1]
        matrix S=subS1,subS2

        * matrix of true probabilities
        matrix Q=.
        sort randtrt2
        by randtrt2: egen psi2mean=mean(psi2)
        replace py=invlogit(alpha + psi1 + psi2mean) if randtrt1!=`meronum'
        forvalues j=1/`ntrtcombs' {
          summ py if randtrt1==S[`j',1]&randtrt2==S[`j',2]
          scalar trueprob=r(mean)
          matrix Q=Q\trueprob
        }
        local ntrtcombsplus1 `ntrtcombs'+1
        matrix P=Q[2..`ntrtcombsplus1',1]

        forvalues j=1/`ntrtcombs' {

          qui replace randtrt1 = S[`j',1]
          qui replace randtrt2 = S[`j',2]
          qui predict pred, rules
          qui replace trueprob = P[`j',1]
          local trt1 = S[`j',1]

          * using no information: random choice (sum true event probabilities for
available treatments to find average)
          qui replace trts = trts+1 if firstcanhave`trt1'
          qui replace EYno = EYno+trueprob if firstcanhave`trt1'

          * using perfect information
          qui replace EYperfect = min(EYperfect, trueprob) if firstcanhave`trt1'

          * using sample information
          qui replace EYsample = trueprob if pred<bestpred & firstcanhave`trt1'
          qui replace bestpred = pred if pred<bestpred & firstcanhave`trt1'

          drop pred
        }
        qui replace EYno=EYno/trts

        egen tagid=tag(id)
        foreach method in no sample perfect {
          summ EY`method' if tagid==1, meanonly
          local EYmean = r(mean)
          gen success`method' = EY`method' < EYperfect + `margin' + 0.0005
          summ success`method' if tagid==1, meanonly
          local successprob = r(mean)
          gen improve`method' = EY`method' < EYno
          summ improve`method' if tagid==1, meanonly
          local improveprob = r(mean)

          post simstemp ("`patterns1'") (`pswitch') (`nobs') (`i') ("`method'") ///
                                  (100*`EYmean') (100*`successprob')
(100*`improveprob')
          }
        }
      }

}
```

```
}

postclose simstemp
}

use `name'_postfile, clear
reshape wide EY success improve, i(i patterns1 pswitch nobs) j(method) string
foreach method in perfect sample {
   gen gain`method' = EYno - EY`method'
}

* generate plots of results
local nobsmin .
local nobsmax .
foreach nobs of local nobslist {
   local nobsmin= min(`nobsmin',`nobs')
   local nobsmax= max(`nobsmax',`nobs')
}
local nobsmax=1.1*`nobsmax'

table nobs pswitch, statistic(mean gainperfect gainsample successsample
improvesample)

local percentmargin=`margin'*100
foreach pswitch of local pswitchlist {
   local percentswitch=`pswitch'*100
   summ gainperfect if pswitch==float(`pswitch'), meanonly
   local gainperfect = r(mean)
   gen gainpercent`percentswitch' = (gainsample/`gainperfect')*100
   meangr8 gainpercent`percentswitch' nobs if pswitch==float(`pswitch'),
name(g_`percentswitch'_`patterns',replace)
saving(g_`percentswitch'_`patterns1',replace) xlabel(`nobslist') xscale(log)
xscale(range(`nobsmin', `nobsmax')) ytitle("% mortality reduction from sample
information" "relative to perfect information") xtitle(Sample size) color(red)
yli(0 100, lcol(blue) lpattern(solid)) lwidth(*2) yscale(range(0 100)) ylabel(#6)
scheme(mrc) note("`percentswitch'% switching to second-line. Reduction from perfect
information: `=string(`gainperfect',"%3.1f")'%")

   meangr8 successsample nobs if pswitch==float(`pswitch'),
name(s_`percentswitch'_`patterns1',replace)
saving(s_`percentswitch'_`patterns1',replace) xlabel(`nobslist') xscale(log)
xscale(range(`nobsmin', `nobsmax')) ytitle("% success" "using sample information")
xtitle(Sample size) color(red) yli(0 100, lcol(blue) lpattern(solid)) lwidth(*2)
yscale(range(0 100)) scheme(mrc) note("`percentswitch'% switching to second-line.
Success: chosen treatment is within `percentmargin'% of best.")

   meangr8 improvesample nobs if pswitch==float(`pswitch'),
name(i_`percentswitch'_`patterns1',replace)
saving(i_`percentswitch'_`patterns1',replace) xlabel(`nobslist') xscale(log)
xscale(range(`nobsmin', `nobsmax')) ytitle("% improving on random choice" "using
sample information") xtitle(Sample size) color(red) yli(50 100, lcol(blue)
lpattern(solid)) lwidth(*2) yscale(range(50 100)) scheme(mrc)
note("`percentswitch'% switching to second-line. Improve: chosen treatment better
than a random choice.")

}

save sims.dta, replace
```

Stata ado file defining the practical_gen function to generate data structure and perform (first) randomisation

```
prog def practical_gen
syntax, Canhave(name) NObs(int) prob(string)

local ntrt 0
```

```
foreach can of varlist `canhave'* {
   local ++ntrt
   local trt = substr("`can'",length("`canhave'")+1,.)
   local trtlist `trtlist' `trt'
}

* treatment subset (pattern)
gen pattern = _n
gen cumprob = sum(`prob')
gen cumn = `nobs' * cumprob / cumprob[_N]
gen n = cumn - cond(_n>1,cumn[_n-1],0)
qui expand n
drop cumn n

* randomise
egen ncanhave=rsum(`canhave'*)
qui gen randtrt=.
gen rand = runiform()
foreach trt of local trtlist {
   qui replace randtrt = `trt' if randtrt==. & `canhave'`trt' & rand<1/ncanhave
   qui replace rand=rand-1/ncanhave if `canhave'`trt'
}
drop ncanhave rand cumprob

end
```

Stata ado file defining the meangr8 function to create graphs of results

```
prog def meangr8
version 11

syntax varlist(min=2 max=2) [if] [in] [, stagger(string) by(string) ///
       overlay missing level(cilevel) BYName ///
    Symbol(string) COLor(string) PATtern(string) LWidth(passthru) Connect(string)
///
    clear debug *]
tokenize "`varlist'"
local y `1'
local x `2'
local graphoptions `options'
marksample touse, novarlist

if "`by'"~="" {
       local byby by(`by')
       local 0 `by'
       syntax varname, [*]
       local byvar `varlist'
       local byopts `options'
}
if mi("`color'") local color navy maroon forest_green dkorange teal cranberry ///
       lavender khaki sienna emidblue emerald brown erose gold bluishgray
       // default for s2color (see http://www.stata.com/statalist/archive/2011-
02/msg00692.html)

* parse stagger
if mi("`stagger'") local stagger 0
cap confirm number `stagger'
if _rc {
       if substr("`stagger'",1,1) != "*" {
              di as error "stagger(#) or stagger(*#)"
              exit 198
       }
       local stagger = substr("`stagger'",2,.)
       local stagger `stagger'*`x'
}


preserve
qui count if `x'==. & `touse'
```

```
if r(N)>0 {
    di in blue "Dropping " r(N) " observations with `x'==."
    qui drop if `x'==.
}
if "`missing'"=="" & "`byvar'"~="" {
    qui count if `byvar'==. & `touse'
    if r(N)>0 {
      di in blue "Dropping " r(N) " observations with `byvar'==."
      qui drop if `byvar'==.
    }
}
collapse (mean) _mean=`y' (sd) _sd=`y' (count) _count=`y' if `touse', by(`byvar'
`x')
local zcrit = invnorm(.5+`level'/200)
gen _upper = _mean + `zcrit'*_sd/sqrt(_count)
gen _lower = _mean - `zcrit'*_sd/sqrt(_count)
label var _mean "Mean of `y'"
local graphoptions note(Showing `level'% confidence intervals) `graphoptions'
if "`overlay'"=="" | "`byvar'"=="" {
    if "`connect'"=="" local c l
    else {
        getfirstc `connect'
        local c = r(firstc)
    }
    if "`symbol'"=="" local s o
    else local s =substr("`symbol'",1,1)
    if "`color'"=="" local color navy
    else local color = word("`color'",1)
    local graphcmd twoway (line _mean `x', lcol(`color') `lpattern' `lwidth') ///
        (rspike _upper _lower `x', lcol(`color') `lwidth'), legend(off) `byby'
`graphoptions'
}
else qui {
    local i = 1
    local go = 1
    while `go'==1 {
        if `i'==1 summ `byvar'
        else summ `byvar' if `byvar'>`min' // levelsof should be used here?
        if r(N)>0 {
            local min = r(min)
            gen _mean`i' = _mean if `byvar'==`min'
            local vallab : label (`byvar') `min'
            if !mi("`byname'") label var _mean`i' "`byvar'=`vallab'"
            else label var _mean`i' "`vallab'"
            gen _upper`i' = _upper if `byvar'==`min'
            gen _lower`i' = _lower if `byvar'==`min'
            local thiscolor = word("`color'",`i')
            if "`thiscolor'" != "" local lcolor lcolor(`thiscolor')
            local thispattern = word("`pattern'",`i')
            if "`thispattern'" != "" local lpattern lpattern(`thispattern')
            local twoway `twoway' (line _mean`i' `x', `lcolor' `lpattern' `lwidth')
///
                (rspike _lower`i' _upper`i' `x', `lcolor' `lwidth')
            local thisorder = 2*`i'-1
            local orderlist `orderlist' `thisorder'
            local i = `i' + 1
        }
        else local go 0
    }
    summ `byvar'
       replace `x' = `x' + (`byvar'-r(mean))*`stagger'
       local graphcmd twoway `twoway', `t1title' `t2title' `graphoptions'
legend(order(`orderlist'))
}
if !mi("`debug'") di as input `"meangr8 is running the command: `graphcmd'"'

`graphcmd'
if "`clear'"=="clear" {
    restore, not
```

```
    global F9 `graphcmd'
    di as text "Graph data loaded into memory: press F9 to recall graph command"
}
end

prog def getfirstc, rclass
if substr("`1'",2,1)=="[" local length=index("`1'","]")
else local length 1
if `length'>0 {
   return local firstc = substr("`1'",1,`length')
   return local rest = substr("`1'",`length'+1,.)
}
else {
   return local firstc = .
   return local rest = .
}
end
```

Stata code defining treatment patterns used in simulations

```
* patterns for first-line randomisation
input canhave1-canhave8
       1  1  1  1  1  0  0  0
       0  0  1  1  1  1  1  1
       0  0  0  0  1  1  0  1
end
gen prob = 1/3
gen alpha = logit(0.20)
save patternsfirstline_1

drop _all
input canhave1-canhave8 prob
       1  1  1  1  1  0  0  0  0.5
       0  0  1  1  1  1  1  1  0.4
       0  0  0  0  1  1  0  1  0.1
end
gen alpha = logit(0.20)
save patternsfirstline_unequal

drop _all
input canhave1-canhave8
       1  1  1  1  1  0  0  0
       0  0  1  1  1  1  1  1
       0  0  0  0  1  1  0  1
       1  1  1  1  1  1  1  1
end
gen prob = 1/4
gen alpha = logit(0.20)
save patternsfirstline_moreinfo

drop _all
input canhave1-canhave8
       1  1  0  0  1  0  0  0
       0  0  1  1  1  0  1  0
       0  0  0  0  1  1  0  1
end
gen prob = 1/3
gen alpha = logit(0.20)
save patternsfirstline_lessinfo


* patterns for second-line randomisation
drop _all
input randtrt canhave1-canhave8
       1  0  1  1  1  1  1  1  0
       2  0  0  1  1  1  1  1  1
       3  0  0  0  1  0  1  1  1
       4  0  0  1  0  0  1  1  1
```

```
       5 0 0 0 0 0 1 1 1
       6 0 0 1 1 1 0 0 1
       7 0 0 1 1 1 0 0 1
end
save patternssecondline
```

Stata code defining treatment effects used in simulations

```
* log odds ratios for regimens as first-line treatment
input randtrt1 psi1
       1 0
       2 -.01
       3 -.17
       4 -.18
       5 -.21
       6 -.28
       7 -.35
       8 -.8
end
gen psi1_rev=psi1[9-_n]
gen psi1_higher25=psi1*1.25
gen psi1_lower25=psi1*0.75
save psi1.dta

* log odds ratios for regimens as second-line treatment
* note treatment 1 (Amp/Pen+Gent) is not used as a second-line treatment
input randtrt2 psi2
       2 -.02
       3 -.15
       4 -.18
       5 -.19
       6 -.21
       7 -.35
       8 -.49
end
gen psi2_rev=psi2[8-_n]
save psi2.dta
```
