# Supplementary material for: Determining sample size in a personalized randomized controlled (PRACTical) trial
Source: Stat Med. Author manuscript; Available in PMC 2024 Oct 1. (PMC7616655; doi:10.1002/sim.10168)
Supplement: Supplementary figures [file EMS198932-supplement-Supplementary_figures.pdf]

Supplementary figures

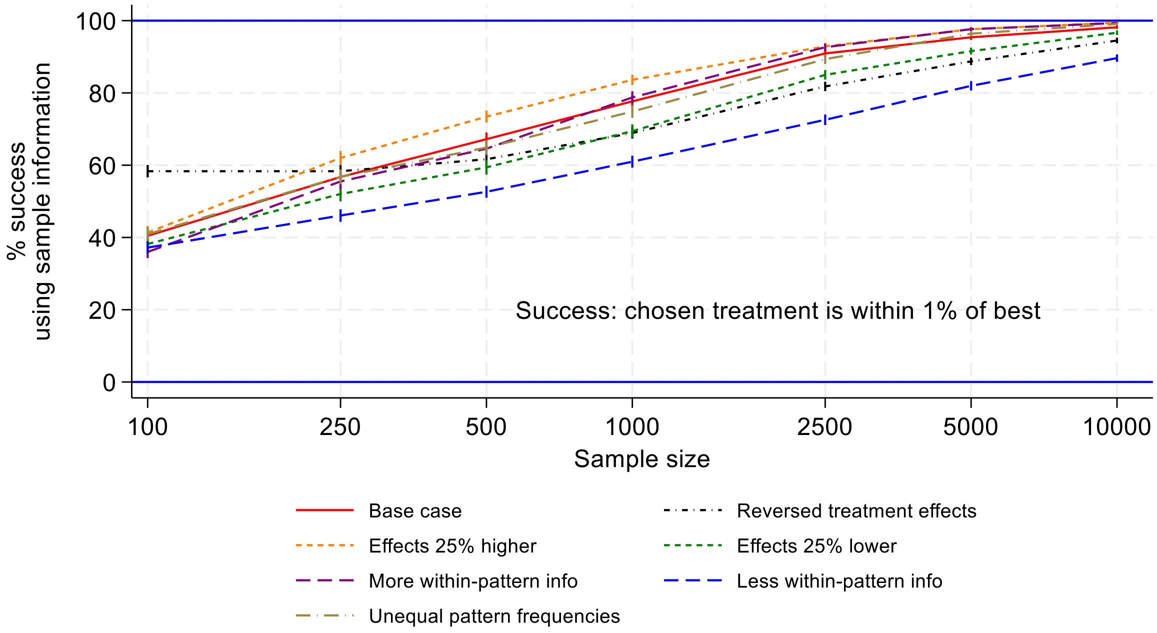

Figure S1 NeoSep1 first-line treatments: Frequency with which the chosen treatment strategy based on sample information was within 1% (in mortality level) of the best treatment strategy

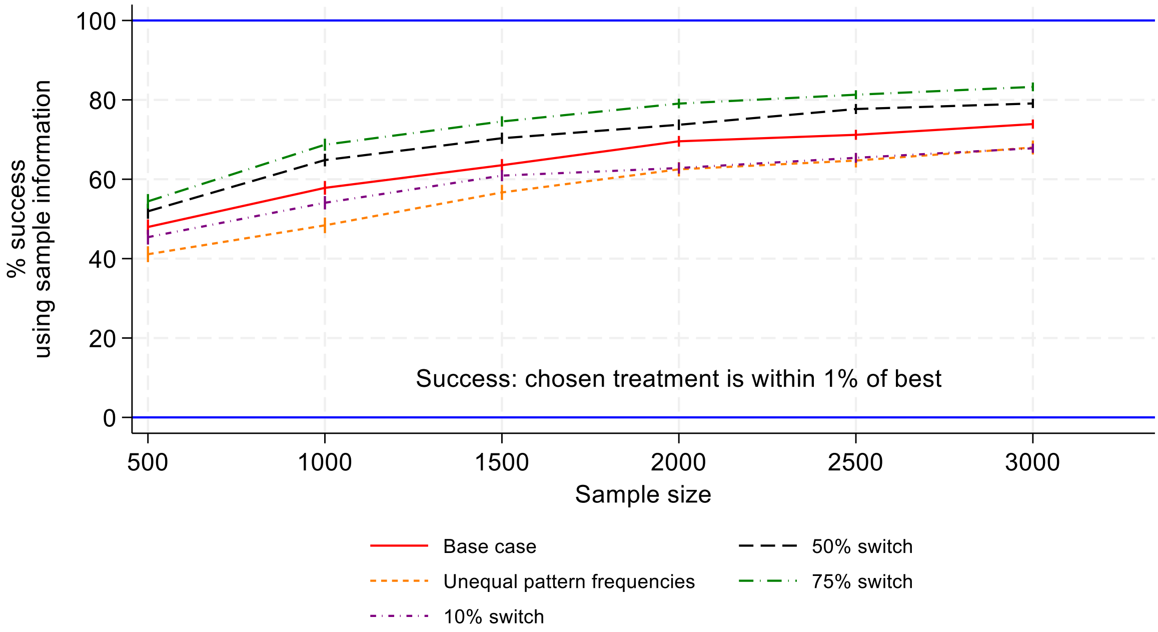

Figure S2 NeoSep1 SMART design: Frequency with which the chosen treatment strategy based on sample information was within 1% (in mortality level) of the best treatment strategy
